# Recommendations for optimising physical activity after gestational diabetes: system targets

**DOI:** 10.1186/s12889-025-25737-y

**Published:** 2025-11-26

**Authors:** Elysa Ioannou, Helen Humphreys, Catherine Homer, Alison Purvis

**Affiliations:** 1 School of Sport and Physical Activity, Sheffield Hallam University, Sheffield, UK; 2 Centre for Behavioural Science and Applied Psychology (CeBSAP), Sheffield Hallam University, Sheffield, UK

**Keywords:** Gestational diabetes, Physical activity, PA, Type 2 diabetes, T2DM, Prevention, Realist, Socio-ecological model, SEM, Systems

## Abstract

**Background:**

Gestational diabetes increases the risk of developing type 2 diabetes ten-fold postnatally, but physical activity can significantly and independently reduce this risk. Yet most interventions targeting women after gestational diabetes have not been able to change physical activity, despite seeing significant dietary changes or weight loss.

**Methods:**

This study used a realist-inspired approach to produce theory-based recommendations, about what could work to optimise physical activity after gestational diabetes. An advisory group was initiated and guided study conceptualisation, recruitment, data collection and reviewed draft recommendations. The socio-ecological model was used to scaffold theories and create meaningful recommendations at different systems-levels. Theories were generated using systematic reviews and grey literature and were further iteratively tested and refined through qualitative data collection with a wide range of professional stakeholders and people with lived experience. Theory-based recommendations were developed and further refined through consultations with women with previous gestational diabetes, researchers, public health professionals, and Diabetes UK representatives.

**Results:**

Ten final theories were generated. Women need to be empowered, feel supported not just depending on family, have access to co-located and affordable childcare in physical activity spaces and be able to share experiences. The final recommendations spanned across social (*n* = 3), organisational (*n* = 6), community (*n* = 3) and policy (*n* = 6) levels of the socio-ecological model.

**Conclusions:**

Fundamental patient care requirements must be fulfilled first, as without improvements in continuity of care and dedicated follow-up appointments after gestational diabetes, there is no opportunity to engage in physical activity conversations. Capitalising on existing community and local resources will also be helpful. To access activity spaces, co-located childcare is essential for some women, with further support to tailor physical activity as needed.

## Introduction

Gestational Diabetes Mellitus (GDM) is a type of glucose intolerance that first appears during pregnancy [1]. Women who have received a diagnosis of GDM are at a 10-times increased risk of Type 2 Diabetes Mellitus (T2DM) after pregnancy [2]. There is general consensus that physical activity (PA) contributes to risk-reduction of T2DM in individuals with impaired glucose tolerance [3–5]. Specifically, the adoption of PA can independently reduce risk of T2DM after GDM by 9% for every 100 min of moderate intensity PA undertaken [6]. Additionally, risk of T2DM after GDM was decreased by 47% for women who increased their PA by 150 min per week compared to women who maintained their PA, even after adjusting for Body Mass Index (BMI) [6].

Despite the benefits of PA after GDM, PA engagement declines at this life stage [7]. This decline has been proposed to result from factors like a lack of childcare [8]. These unique barriers to PA postnatally are also not addressed in interventions that target women after GDM [9]. Furthermore, it has been recognised that PA is not effectively encouraged after GDM [10]. However, at the time of conception of the proposed work in this manuscript, there was no evaluation, theory or understanding based on these interventions and outcomes about what could optimise PA engagement after GDM. Therefore, how to best support PA after GDM needs to be investigated.

Pawson and Tilley developed the concept of a realist evaluation to explain why and how interventions result in a given outcome [11]. Understanding what works, for whom and in what contexts could improve uptake in groups of people who may otherwise not engage [12]. In the case of women after GDM, this approach could help to explain why some women engage with PA and others do not, and how to subsequently optimise PA engagement. Realist And Meta-narrative Evidence Syntheses: Evolving Standards (RAMESES) have published guidance to improve the quality of conducting and reporting of realist evaluations and reviews [13, 14]. These detail strict methods, specifically geared towards understanding contexts and mechanisms around one particular intervention or program.

However, a ‘systems’ focus for behaviour change [15] could be more effective in terms of how to optimise PA after GDM. Specifically, multi-level interventions could be more effective for improving population health outcomes compared to ‘single-level’ interventions, which may otherwise widen health inequalities [16, 17]. This is because individual behaviours do not happen in isolation, with cultural, social and other contextual factors largely determining health behaviours [18]. A socio-ecological approach highlights the need to include these multi-level factors, in addition to the intrapersonal factors affecting behaviour, when trying to change behaviour. This approach may be more effective for women with previous GDM to be active in the long-term, considering the interacting social, environmental and policy factors impacting PA and could further identify where solutions should be implemented [19–21]. The Socio-Ecological Model (SEM) is a theoretical framework that visually displays interrelationships between social, physical, and policy environments surrounding and impacting individual health behaviour [22]. The SEM is thus a useful tool for identifying, organising and framing multi-level opportunities for optimising PA. Therefore, to understand what could work and understand why a range of interventions have not yet successfully targeted PA, this study proposes a realist-inspired approach that incorporates this ‘systems’ perspective, described further below.

## Methods

### Aim and objectives

The overall aim of the study was to produce pragmatic, theory-based system-level recommendations about what could work to optimise PA after GDM. The objectives were:

i. Systematically review the literature to understand what interventions targeting PA after GDM exist, and which (patterns of) components of these most likely support PA.
ii. Explore grey literature and further qualitative studies to understand the barriers and facilitators to PA, to better understand why the intervention components identified in aim (i) may or may not be more likely to support PA.
iii. Develop initial theories about what could work and what is not working to support PA after GDM.
iv. Test these theories using qualitative data collected with women who have had GDM, healthcare professionals and other stakeholders.
v. Develop final theories about what could optimise PA, using the SEM as a framework to direct where targets would be best placed.
vi. Produce recommendations for practice based on the final theories.
vii. Refine recommendations through consultations with a range of stakeholders to develop clear system-level targets for optimising PA after GDM.

## The researchers and context

This study is an overview of the process the lead author, EI, undertook for her PhD thesis. To note, the initiation of the project was in February 2021, with the final analysis and write-up concluded by April 2024. EI was a PhD student with a background in sports science and a registered nutritionist at the time the study was conducted and is currently a researcher on an NIHR funded project. HH, CH and AP were supervisors of the lead author. HH and CH are experienced qualitative researchers and public health practitioners with qualifications in exercise and health psychology.

### The advisory group

An advisory group was initiated at the start of the research process. The purpose of this group was to ensure the research was relevant, acceptable and better communicated [23]. Women were eligible if they had a previous diagnosis of GDM. Recruitment occurred through; (1) connections already established through an online Diabetes UK GDM support group that was offered during the COVID-19 lockdown, (2) word of mouth and (3) through a poster shared on social media. Women who were interested in being part of the advisory group were sent an information sheet detailing potential involvement in the project over the three-year period. To note, while not strictly monitored, most members of the advisory group were recruited through the Diabetes UK, with a few additional individuals recruited via word of mouth. The social media advert generated minimal engagement. Therefore, establishing connections within relevant community groups may support recruitment of advisory groups for research purposes.

**Fig. 1:**
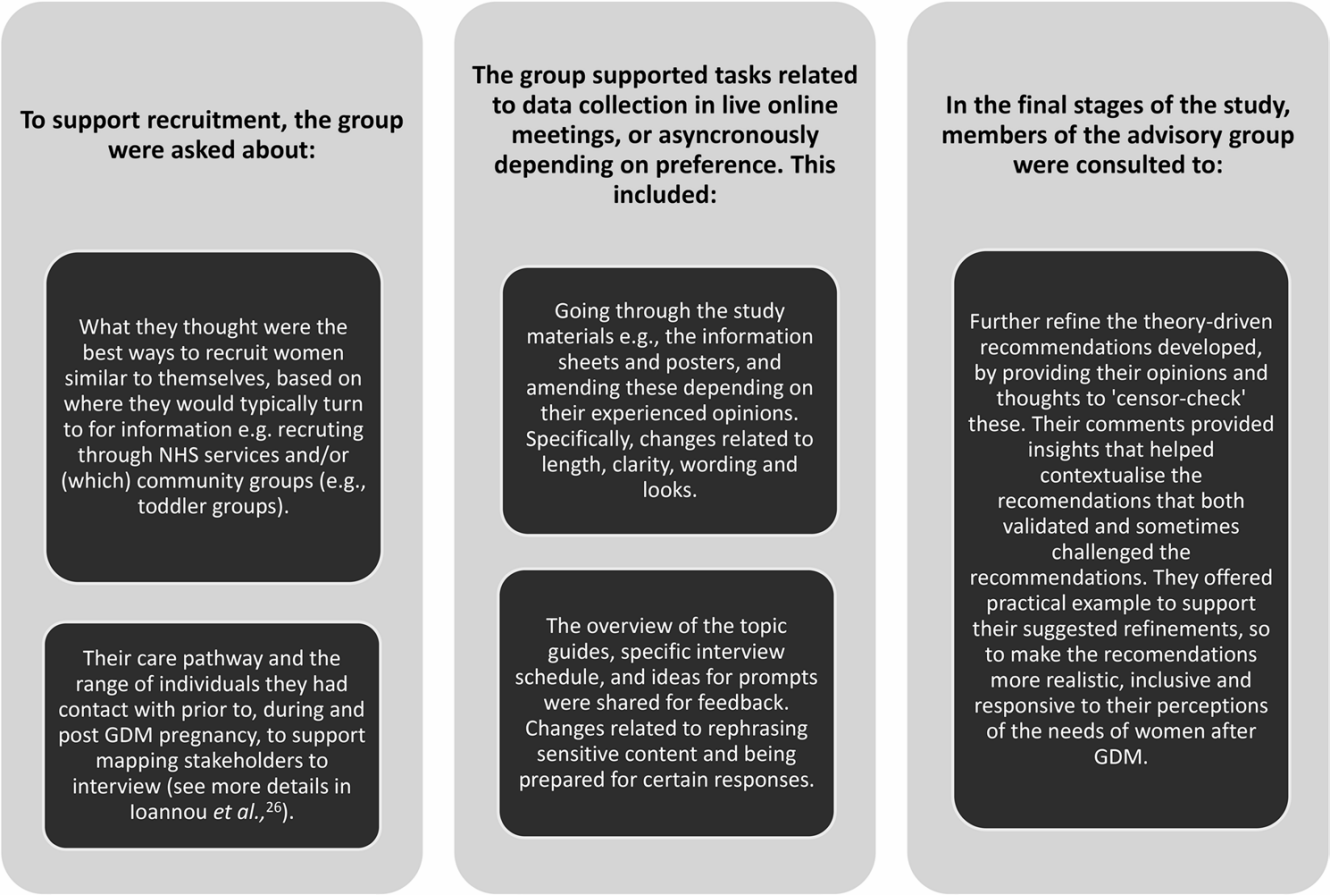
Summary of the advisory group’s involvement throughout this study

A total of eight women were in the advisory group for the duration of the study. Demographic and personal information was not collected, as the eligibility to be in the advisory group was to have had a previous diagnosis of GDM. They were contacted periodically with updates about the research, and a clear opportunity to opt out of the group and email updates if they no longer wished to be involved. Where their input was needed, they were emailed and offered remote one-to-one meetings according to their schedules. The ability to flexibly dip in and out of meetings was key for these women, who stated this approach made their involvement with the project easier and less stressful. Not all women were able to attend all meetings with the lead researcher over the study duration. Between three to five women were involved at each stage of the consultations, outlined in Fig. 1. They were reimbursed for their time and contributions during meetings, as per NIHR guidance on payment for involvement [24].

### The proposed theoretical framework: the Socio-Ecological model

As described above, the SEM was employed in this work as a useful tool for framing the ‘systems’ surrounding women after GDM, and to better make sense of the potential ‘multi-level’ targets to support sustained behaviour change i.e., PA. Definitions of the ‘levels’ within the SEM are not definitive and can be open to interpretation. Therefore, for clarity and transparency, we report a detailed description of how the SEM was implemented in this work below.

## How the SEM was used

In this study, women after GDM were central to the ‘individual’ level. Components within other levels were categorised depending on how they were thought to interact or impact women after GDM. See Table 1 for a summary of how the levels of the SEM were defined and applied in this body of work.

**Table 1:** Summary of how each level of the SEM was defined in this study, with examples

Level of SEM	Description	Example
Individual	Specifically, the individual level was defined as any influence directly on the individual like knowledge, beliefs and attitudes.	For example, motivational interviewing targets individual motivation to PA.
Social	The social level was defined as any social interaction or influence on behaviour by surrounding contacts such as HCPs, friends, family or partners.	For example, targeting partner beliefs by including them in education sessions.
Organisational	The organisational level was used to define contexts influential on PA or organisation-based contexts within the lives of postnatal women after GDM.	For example, anything related to or based within healthcare settings was categorised at this level.
Community	The community level was defined in two separate ways. 1. The first was in reference to physical locations or gatherings, such as community-based support groups or local resources. 2. The second way the community level was defined was as how people are linked by shared experiences like religion, culture or others.	For example, for postnatal women after GDM, support groups as ‘mum and baby’ groups were categorised at the community level.
Policy	Finally, the policy level was used to categorise initiatives and guidelines, either locally or nationally, that impact women who have had GDM	For example, national diabetes prevention initiatives, PA guidelines, or funding decisions.

*PA* Physical Activity, *HCP’s* Health Care Professionals, *GDM* gestational diabetes mellitus, *SEM* Socio-Ecological Model

Initially, theories were aligned within specific levels of the SEM. As the iterative rounds of refinement progressed, it became clear that theories acted across and within levels of the SEM. The complexities of the system within which each theory operated would be ignored by forcing and categorising these within distinct levels of the SEM. On reflection, this further highlights how the levels of the SEM are not discrete, and how the SEM is simply a framework that can guide organisation of thoughts and theories. Instead, in this study, multiple levels of the SEM were framed and addressed within each theory, to truly capture a multi-level explanation and approach for a solution. Theories were thus presented in a stand-alone manner, with how they may operate within different SEM levels presented separately. Recommendations were developed based on the theories around what could work to optimise PA. These recommendations were framed within levels of the SEM, to provide an overview of where targets may lie.

### The realist-inspired approach

As described in the introduction, a strictly realist approach or evaluation is well suited to one intervention or program [9, 10]. However, the aims of this work were to understand ‘what works’ across different ‘systems’, towards a whole-systems approach to support PA after GDM. Therefore, the approach was classed as ‘realist inspired’ – meaning realist methods were employed but adapted to suit a more ‘systems’ thinking approach. Further details of the realist-inspired approach are below.

In a realist approach, theories are generated to explain the ‘mechanisms’ leading to observed outcomes/phenomena^25^. Thus, in the realist-inspired approach implemented, this core concepts remained. The process of scaffolding theory onto an existing appropriate theoretical framework is also a realist method [26], however in the realist-inspired approach implemented, this theory was not an intervention program theory and instead was based on a socio-ecological framework. Additionally, while theories were generated and presented as ‘IF-THEN-BECAUSE’ statements, the ‘mechanisms’ presented were not strictly mechanisms relating to a specific program [27]. Instead, mechanisms presented in this study were proposed to explain what might work for women after GDM to better engage with PA (BECAUSE), depending on the context (IF) and outcome (THEN). This format i) suits the pragmatic lens of the study, ii) utilises more accessible language for policy and practitioners who form the target audience of this work, and iii) has been previously used as a practical way to generate and present theories [28].

**Fig. 2:**
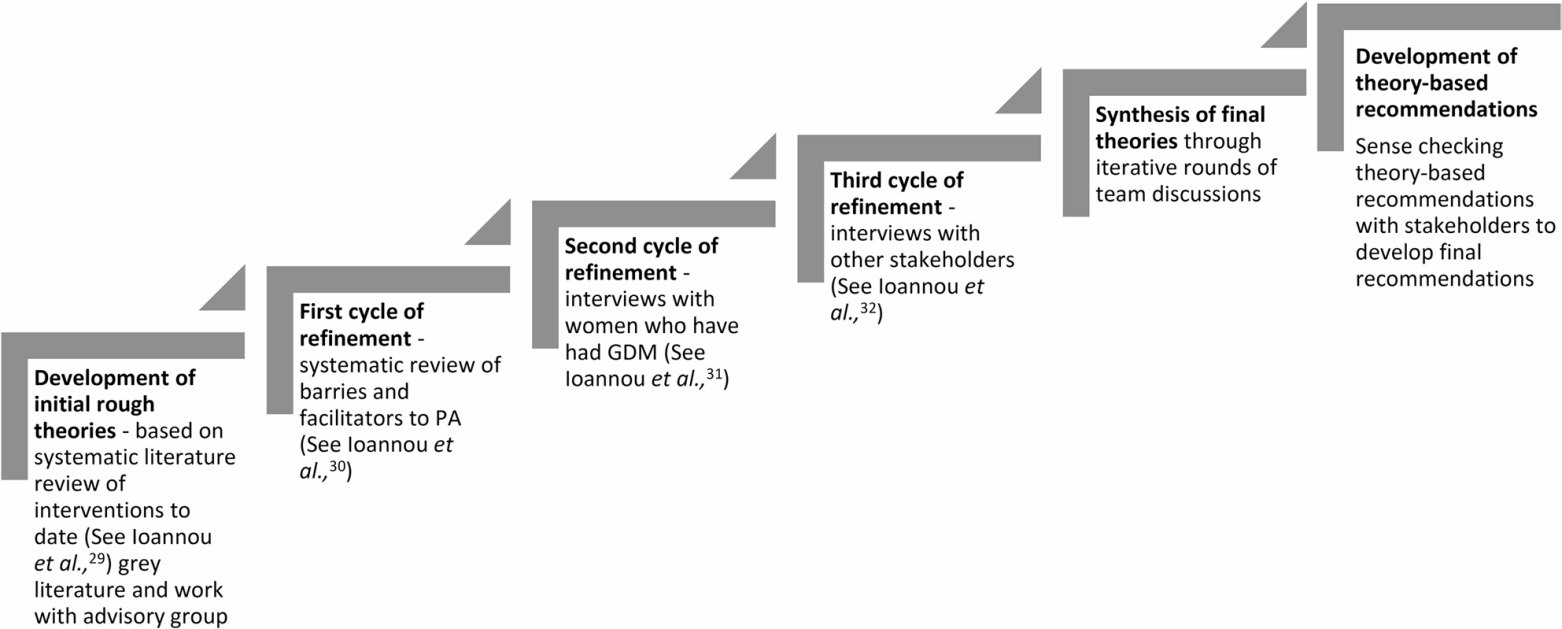
Visual display of cycles of refinement for theory development

Figure 2 presents an overview of how several studies and rounds of refinement contributed to the realist-inspired theories presented in this manuscript. While Fig. 2 displays these stages as discrete and in sequence, in reality the process was iterative, going back and forth through these stages as theories were refined. Additionally, data from a range of sources and methods were used to build, test and refine the theories, as is suggested with realist research to better understand what works, or what does not work [13]. The process of refining theories was aided further through discussions within the study team, the advisory group and other stakeholders.

Specifically, initial theories were generated by first systematically gathering all interventions that targeted PA after GDM (now published [29]), to gain an understanding of what had been done. The systematic review investigated components employed in interventions and analogous PA outcomes. Sister papers, any fidelity work, grey literature and others were then considered in the initial theory-building process, to try and understand and explain the PA results observed in these studies, depending on what tools and techniques the studies employed. The logical next step to test, refine or refute these theories involved another systematic literature review [30], to understand the barriers and facilitators to PA that could further improve understanding of the mechanisms of what could work to optimise PA engagement. This helped better understand what was or was not working within the interventions. Theory development was further supported through developing ‘IF-THEN’ statements [28]. Specifically, patterns and trends were identified and written as ‘IF’ statements. These were linked with results to form analogous ‘THEN’ statements.

At this point, the refined theories were further tested through primary data collected, collected with women [31] and then with healthcare professionals [32] by explicitly and directly seeing if there was any agreement or disagreements with these theories which also provided more information on the context. Ethical approval was obtained prior to any data collection, which spanned from September 2022 until December 2023, specified further in Ioannou et al.,^31,32^. The interviews included direct questions to test theories developed up until that point, which is considered a useful way to refine theories [33]. Final theories were then generated through discussions with the advisory group and the study team in iterative rounds spanning four months of meetings. This included the lead author meeting, having discussions, collating information, refining the theories and then doing this all over again until the theories were finalised. The outcome included ideas of what was working, what was not working and for who and why this was the case. Subsequent recommendations built upon these empirically tested theories. These recommendations and refinement of theories were based on the SEM, described further below.

### The development of theory-based recommendations

The realist inspired theories generated to understand what could work and why for optimising PA were used to develop theory-based recommendations. As described above, these initial recommendations were generated by the lead author (EI) and refined through rounds of reflexive discussions with the remaining authors (HH, CH, AP). They were then displayed where they could best be targeted according to the SEM. These initial recommendations were then refined and sense-checked amongst a range of stakeholders and the advisory group to develop final recommendations.

In total, nine stakeholders were consulted to provide feedback on theory-driven recommendations. This group included three women from the advisory group established at the start of the PhD who had previously experienced GDM, two researchers specialising in postpartum and/or GDM-specific women’s health topics, two stakeholders involved in public health and/or policy, and two professionals from Diabetes UK. Six discussions were held over Zoom, each lasting about 60 min, while three stakeholders provided feedback through written comments via email. During the live online conversations, a summary was presented outlining the origins of the recommendations, including a description of the data collection and theory generation processes that led to their development. Each recommendation was presented individually, allowing time for comments, thoughts, and feedback, which were recorded in a separate document. The feedback was then compiled, and the final recommendations were edited and refined. The SEM levels were used to structure these recommendations, with each level indicating the target area for each recommendation.

## Results

As highlighted in Fig. 2, the evidence leading to the theory generation has been separately synthesised and published elsewhere [29–32]. Table 2 presents the ten final theories generated. Addressing just one theory will not necessarily ‘work’ or result in increased PA. Instead, multiple theories across levels are needed, for whole systems approaches to optimise PA in women after GDM. Targets as to what may work in accordance with the theories across the SEM were further considered and are presented in Table 3. Theory-driven recommendations are subsequently presented in Table 4.

**Table 2:** Final theories to understand what could work to optimise PA after GDM

Theory title	IF	THEN	BECAUSE
*Theory 1*: *Women need to feel empowered to engage with physical activity after gestational diabetes.*	Women feel able and want to engage in PA	They will try and find a way to make PA realistic and achievable	They feel driven and empowered to overcome obstacles they believe they can overcome
*Theory 2*: *Weight-loss or weight management as motivating factor for physical activity can be highly variable.*	Interventions or services have a weight-focus	PA outcomes will be variable	Women are differently motivated by weight-based outcomes
*Theory 3*: *Support from partner*,* significant other or wider family enables physical activity.*	Women receive encouragement and physical support from their wider family or partner/husband/wife	They may find engaging with PA is easier and seen as positive and beneficial	Shared understanding of positives of PA between partners, may help women feel less guilty, nudged to be active as they feel it is worth it, plus childcare responsibility is removed.
*Theory 4*: *Professionals and/or practitioners need to provide personalised support for physical activity in women after gestational diabetes.*	Women have professional support encouraging PA	They are more likely to listen and take on board PA advice	They have respect for the authority figure, so have better buy-in and engage with suggestions for PA
*Theory 5: * *Co-location of affordable and beneficial childcare in physical activity spaces is essential to access physical activity after gestational diabetes.*	There is no childcare provision at the venue for diabetes education or purposeful exercise,	Low turnout or adherence to intervention, due to inability to attend.	Women are unable to take time and attend the venue and/or program, due to childcare responsibilities.
*Theory 6*: *Women with previous gestational diabetes who feel guilty taking time to engage in physical activity may be less inclined to do so.*	Women think PA negatively impacts their ability to undertake their ‘role as a mother’	They will not prioritise engaging in PA	They may experience guilt in prioritising themselves or PA and feel like they must, instead, prioritise looking after their children. Where PA/intervention is not considered a priority in comparison to other demands, focus and time goes to children, household chores or work instead, so no time to focus on themselves or engage in PA.
*Theory 7*: *Location*,* cost and access to physical activity overshadows and constrains women’s desire to engage with physical activity.*	If women cannot afford or access flexible, affordable PA	They will be unable to engage with PA	The barriers to accessing PA are outside of individual control. Despite potential desire to engage, it is too difficult to do this. Either PA is too costly, or too difficult to access. Where PA is located further away, time to access becomes a barrier.
*Theory 8*: *Poor continuity of care for women after gestational diabetes reduces contact points and opportunities for physical activity conversations.*	HCPs don’t have contact with women after GDM	They are losing the opportunity to engage in PA discussions, refer to PA schemes and refer into NDPP	Women may be unaware of increased risk of T2DM or may not perceive this as a pressing issue, or not in the position and not feel supported to be more active.
*Theory 9*: *Connecting women after gestational diabetes can reinforce and facilitate collective physical activity.*	Women are connected after GDM and/or have group-based PA settings	Women are more likely to feel supported and engage in PA	Allows for social relatedness, peer support and provides opportunity to share experiences and feel heard. Women may also feel supported for PA through accountability and shared motivation to engage with PA and desire to help each other to do so.
*Theory 10*: *Physical activity resources within communities and locally are valuable assets that should be mobilised in supporting women after gestational diabetes.*	Free or cheaper (council-led) PA schemes, initiatives and resources exist	Women may be more likely to access these resources	There is greater opportunity to access PA when opportunities are within communities or localities, may make them easier to access, and women may be more likely to engage with these resources because of familiarity, ease, affordability, and comfort in doing so.

*GDM* Gestational Diabetes Mellitus, *PA* Physical Activity, *T2DM* Type 2 Diabetes Mellitus, *HCPs* Health Care Professionals, *NDPP* National Diabetes Prevention Program, *DUK* Diabetes UK

**Table 3:** How final theories apply within different levels of the SEM to optimise PA after GDM

Considerations at SEM level				
Intrapersonal	Social	Organisational	Community	Policy
*Theory 1*: *Women need to feel empowered to engage with physical activity after gestational diabetes.*				
Changing mindset, addressing individual motivation, readiness and self-confidence is an internal mental process for women after GDM	Coach HCP or other individual will be implementing BCTs with women after GDM.	Resources, interventions and support based in healthcare or other organisations.	-	Policy must train and direct resources for HCPs to learn and incorporate a compassionate-informed approach.
*Theory 2*: *Weight-loss or weight management as motivating factor for physical activity can be highly variable.*				
Addressing beliefs about PA, or self-worth related to body-image.	Coach, HCP or other individual detangle PA promotion from weight-based approach.	-	Shifts in societal norms and thinking around PA solely for weight goals.	-
*Theory 3*: *Support from partner*,* significant other or wider family enables physical activity.*				
Feeling supported by partners or family members could increase perceived capacity and capability for PA.	Partners and family members are the immediate social support around women after GDM.	Availability of appropriate childcare opportunities (that are affordable) would overcome the difficulties associated with relying on family for childcare to enable PA.	-	-
*Theory 4*: *Professionals and/or practitioners need to provide personalised support for physical activity in women after gestational diabetes.*				
Target individual motivation and perceived capacity and capability for PA.	HCPs or teams implementing interventions provide counselling and/or social support for women after GDM.	Training of HCPs by making use of the structure and training capacities existing within healthcare. Provision of support through organisations.	-	Policies to require training of e.g. compassionate approach and allocate funding and resources to increase support available.
*Theory 5*: *Co-location of affordable and beneficial childcare in physical activity spaces is essential to access physical activity after gestational diabetes.*				
-	-	Organisations e.g. gyms to implement co-located childcare opportunities within facilities.	-	Policies around childcare provision or subsidised childcare opportunities.
*Theory 6*: *Women with previous gestational diabetes who feel guilty taking time to engage in physical activity may be less inclined to do so.*				
Changing personal held beliefs about what being a ‘good mum’ looks like.	Education and support to overcome guilt associated with prioritising oneself, carried out and implemented by e.g. partners, HCPs etc.	Impacted by the workplace (and organisations including for child enrichment) which demand time and prioritisation. Flexible access to PA and PA support to be implemented by organisations.	-	-
*Theory 7*: *Location*,* cost and access to physical activity overshadows and constrains women’s desire to engage with physical activity.*				
Providing information and access to information and how-to’s. Women may need further support for motivation and capability to use information provided.	-	Improve access through providing information for home-based PA, the location of e.g. gyms and other organisations that offer PA opportunities, and the cost to access these.	Schemes, referral opportunities and community-based PA that may exist already to be made use of.	Goal of getting people active on a population and public health level for the availability and funding of exercise referral schemes or free- at-point-of access PA opportunities based locally.
*Theory 8*: *Poor continuity of care for women after gestational diabetes reduces contact points and opportunities for PA conversations.*				
Linking with BCTs, providing information and guidance for how and what PA to undertake, even if not perfect.	Increased contact points and time with HCPs to increase postpartum support.	Continuity of care, especially the transition from birth back to primary care. Roles for PA specific support to be situated within healthcare or other organisations.	-	National initiatives requiring follow up to improve continuity of care and recall systems. Policies directing resources and funding maternal health services. Adaptations to the NDPP.
*Theory 9*: *Connecting women after gestational diabetes can reinforce and facilitate collective physical activity.*				
-	Support and connecting with other women, PA encouraged with social support and socialising during PA.	Hybrid approach to overcome access issues with group constraints and timings. Supporting charities and other groups to initiate support groups for women during and continuing after GDM.	The need for and social relatedness and bonding with other women in group-based settings. Connecting women with a shared experience to foster a sense of community, connection, and support.	-
*Theory 10*: *Physical activity resources within communities and locally are valuable assets that should be mobilised in supporting women after gestational diabetes.*				
-	HCPs in contact with women to be trained and educated about other schemes and resources that women after GDM may be eligible for and benefit from.	Directing to appropriate resources at the right time linked with appropriate organisational structures ensuring continuity of care and contact with women after GDM. Also related to the availability and access to resources and schemes.	Ensuring opportunities are based in communities, e.g. gyms, or community hubs. Or making use of existing communities e.g. religious settings.	Funding of (wider) opportunities and PA schemes, directing resources must occur nationally, and locally, within government and local councils.

*GDM *Gestational Diabetes Mellitus, *PA* Physical Activity, *T2DM* Type 2 Diabetes Mellitus, *HCPs* Health Care Professionals, *NDPP* National Diabetes Prevention Program, *DUK *Diabetes UK, *BCTs* Behaviour Change Techniques including educating about diabetes risk, goal setting, motivational interviewing, self-monitoring, using reminders and providing feedback

**Table 4:** Summary of recommendations with explanation, examples and indications of which theories contributed to their development

Recommendation	Stakeholder group	Explanation and Example	Theories
Social level			
Women after GDM must be supported postnatally and counselled to: 1) Overcome ‘mum guilt’. The benefits of PA must be emphasised, educating women that taking time for PA doesn’t make a parent ‘bad’ or ‘selfish’. 2) Promote PA positively, disentangling sole emphasis on PA for weight-loss and encouraging imperfect action (something is better than nothing). 3) Provided with ideas and instructions for exercise (how-to’s) and be supported to find acceptable and varying forms of PA, that works within their lifestyle, time, space, and access available.	Professionals and practitioners working with women after GDM e.g. HCPs, NDPP practitioners, implementation of PA interventions to ensure supportive counselling for PA.	Women are individuals within complex interacting systems that impact and constrain individual choice. Interventions after GDM all incorporate BCTs, but do not give structured PA support, with women after GDM wanting more information and advice on types of and how to undertake PA. Role as mother and guilt were also important determining themes for PA that need to be addressed to improve PA uptake. This recommendation is placed at the social level, as it is support from professionals interacting with women that would implement this recommendation. Training of HCPs and other stakeholders necessary to achieve this recommendation would be placed at the organisational level. Further training in using a compassionate approach and patient-centric advice could also be beneficial. For example, where access to a gym or other PA opportunities may not be available, ‘how-to’s for exercise could include realistic workout guides and exercises that can be done within the home. Where space is an issue, this could be suggesting outdoor based PA or working collaboratively with women to identify alternative ways to incorporate a significant increase in PA throughout the day.	1, 2, 4 and 6
Partners of women after GDM should be included in planning realistic PA and discussions around lifestyle and managing T2DM risk.	• Partners/significant others • Organisations and charities working with women after GDM e.g. HCPs, NDPP, DUK to allow and encourage partners within discussions.	Partner support is important to enable women after GDM to enact lifestyle changes including PA. Partner support is mainly needed to address a lack of childcare. Including partners in PA planning may aid with this process. However, ideally, the inclusion of childcare co-located in PA spaces may be a more inclusive way to enable PA, especially for women who may not have partner or wider family support. For example, interventions aiming to increase PA after GDM should include partners in goal setting and planning exercises with women, to enable planning of realistic opportunities for PA.	3, 5 and 6
***Organisational level***			
Childcare should be co-located in PA spaces that must be enriching, flexible and affordable. PA opportunities for women should also be co-located in child-focused spaces.	• Academics in intervention planning to co-produce and evaluate the acceptability of co-locating childcare with PA opportunities. • Local councils and local government to fund and/or provide affordable opportunities for co-located childcare in PA spaces.	Childcare is widely recognised as a barrier to PA for women after GDM. Immediately postpartum mum and baby opportunities exist, but a gap in opportunities in the extended postpartum period was highlighted. For this reason, the co-location of childcare opportunities could be key to enabling the uptake of PA after GDM. Examples and evaluations of co-located childcare opportunities in PA spaces are lacking. Future research should examine the effectiveness and implementation of co-located childcare for PA engagement. For example, council-based community leisure centres could offer crèche facilities that are cheap for women to be able to use while they use the leisure-centre facilities. Children’s activities e.g. sports teams could have a branch or arm for women to be active while their children are participating in said activity.	5,6 and 7
Flexible access to PA and/or other programs, should be prioritised, including utilising hybrid approaches, incorporating live and remote sessions and allowing children to be in sessions.	Academics and public health practitioners in intervention planning to investigate and evaluate remote live sessions and how best to increase the flexibility of counselling and support for PA.	Face-to-face options were important for support and engagement yet could be challenging to access. Fully remote, asynchronous content was not thought to be helpful on its own, with more interaction needed. Remote sessions were suggested, as long as they were live, to improve flexibility to access. For example, interventions containing counselling and support for PA could include live video consultations to aid flexibility in attendance.	4, 5, 6 and 8
***Community level***			
Form support groups and connect women after GDM for peer support to enable exercise through: 1) exercising together with their babies/children and 2) through keeping each other accountable for exercise and PA.	Organisations and charities working with women after GDM e.g. HCPs, NDPP, DUK to connect women after GDM together.	Some women preferred to exercise in group settings or have peer support and encouragement to be active, forming a ‘buddy system’ to encourage exercise. Sharing this with women who were in similar positions and situations was important for feeling understood through shared experiences rather than judgment or shame. For example, NDPP sessions could group women after GDM together, rather than mixing with an average, potentially older population that may otherwise be referred to the NDPP. Another example could be antenatal teams connecting women after GDM to charities, such as DUK, which could run local support groups for women during and after GDM. These groups could be multifaceted, sharing experiences, including education around managing future T2DM risk and encouraging women to be physically active together e.g. buggy walks in this group setting.	8 and 9
Free (or cheap)- at-point-of-access PA opportunities within communities and/or locally based (by neighbourhood or locality, through religious settings, sports teams, schools, children centres/family hubs etc.).	• Local councils and local government to fund and prioritise accessible and free or cheap at point of access PA opportunities. • Organisations and charities working with women after GDM e.g. HCPs, NDPP, DUK to connect women to these opportunities. • Charities and organisations that run spaces within communities e.g. religious settings, children’s centres etc.	Cost and distance to access PA was a barrier to PA. Links to community-based or locally accessible PA opportunities could make use of existing, more accessible PA resources. Therefore, where PA initiatives and schemes may exist that are council-run or lead, healthcare or other community organisations should link to and direct women after GDM to these opportunities and resources. This may require training and advertising of such opportunities to increase reach. For example, antenatal teams, or where possible postnatal contact e.g. through primary care should direct women after GDM to council-run gyms offering affordable or means-tested memberships to aid access to PA within local settings. Exercise classes for new mothers could also be based in children’s centres or family hubs. This would require funding and set up, ensuring PA opportunities, instructors and professionals be based out of centres, making this sort of PA free or cheap to access and suitable for women at different stages of the postpartum journey.	7 and 10

**Table:** 

Policy level			
Funding and providing resources for a dedicated role within healthcare contexts to PA and supporting lifestyle behaviours.	• Policy makers and public health practitioners to allocate funding and create role. • Primary care to embed Sport Scientists and PA specific qualified individuals. • Academics to evaluate feasibility and cost-effectiveness.	While it is important for HCPs in primary care to encourage PA, stakeholders highlighted the lack of time and need to focus on other issues during appointments. There was discussion about using social prescribers to have longer more detailed conversations around PA for women after GDM. Women after GDM also discussed that having access to exercise professionals was valuable for specific PA discussions. Healthcare structures should embed and incorporate PA specific support. For example, PA specialists qualified in exercise prescription should be embedded within GP surgeries for a range of conditions. Women after GDM should be automatically referred to these specialists for in-depth counselling and support to aid in engaging with PA postpartum that is realistic for them, and to further support a transition towards lifelong PA.	1, 4 and 8
Policies, funding and resources directed to ensuring follow up at/after 3-months postpartum, for the transition away from GDM into discussion and introduction to long term diabetes risk management.	• Public health practitioners to require a 3-month follow up as part of the GDM and post-GDM support pathway and consider incentives for GPs to prioritise this group. • Primary care should send reminders and prompts for follow up after GDM.	Discussion of the best timing for PA was refined over the course of this study, which, while the appropriate timing is specific to individuals, there must be postpartum follow up. Preferably, this should occur after the initial 3-month transition period, but also be available later on for women to engage with when they are ready. There could be several points where prompts and primary care may try follow-up with women post-GDM, for example and 3, 6 and 12 months, whereby further support for lifestyle and transitioning behaviours away from pregnancy specific GDM management and into behaviours important for T2DM risk should be counselled, including dedicated support for PA. This support is necessary as part of continuing care post GDM and reducing future risk of T2DM, in part through support and directing to local resources for PA. Reducing loss at follow up could be productive e.g., by: • Official discharge from the hospital after birth to aid the application of read codes in GP practices. • Allowing the antenatal team to make referrals to the NDPP. • Funding of and implementation of a recall system within GP surgeries, ensuring women after GDM are invited for annual screening. Screening appointments can be well placed to point women after GDM in the direction of PA schemes within communities and/or the NDPP.	1, 4 and 8

*GDM* Gestational Diabetes Mellitus, *PA* Physical Activity, *T2DM* Type 2 Diabetes Mellitus, *HCPs* Health Care Professionals, *NDPP* National Diabetes Prevention Program, *DUK* Diabetes UK, BCTs Behaviour Change Techniques including educating about diabetes risk, goal setting, motivational interviewing, self-monitoring, using reminders and providing feedback

### Final recommendations

**Fig. 3:**
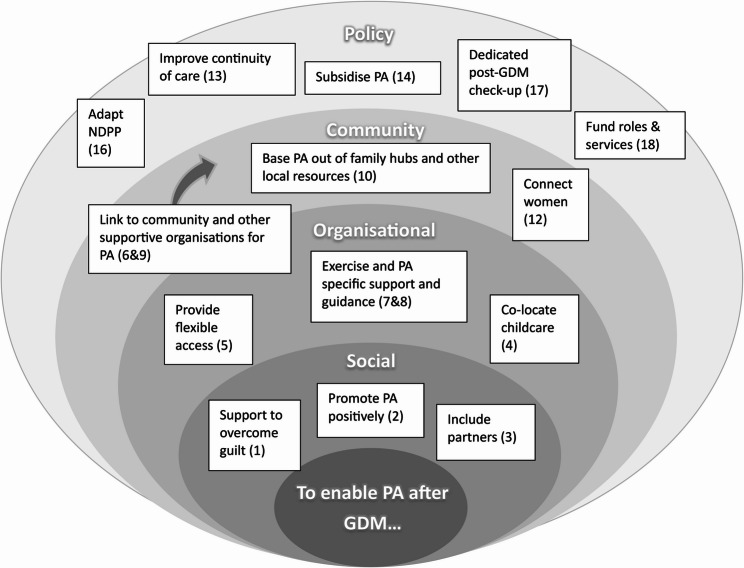
Final recommendations mapped to SEM targets to optimise PA after GDM

The final recommendations to enable women after GDM to engage with PA are presented below. The difference in the recommendations presented in Table 4 versus the below reflects the changes made after stakeholder feedback was gathered. See Fig. 3 for a visual and key-word summary of the final recommendations post-discussion with stakeholders.

### Social

#### HCPs e.g., health visitors, GPs etc., and other relevant stakeholders should….

1. Help women overcome ‘mum guilt’, emphasising the benefits of PA and educating that taking time for PA doesn’t make a parent ‘bad’ or ‘selfish’.
2. Promote PA positively, disentangle PA promotion from ties to weight-loss and encourage imperfect action i.e., something and any movement is better than nothing.

#### Partners should…

3) Where possible, appropriate, and deemed safe, partners should be included in planning realistic PA and discussions around managing T2DM.

### Organisations

#### PA opportunities e.g., gyms, exercise classes, walking groups etc., need to…

4) Prioritise co-located childcare opportunities (and/or Crèche services) that are enriching, flexible and affordable.
5) Enable flexible access, including utilising hybrid approaches and incorporating live and remote sessions.
6) Be affordable e.g., through council-led initiatives and subsidising costs.
7) Provide exercise and PA specific support and guidance. Support could involve ideas and instructions for exercise (how-to’s) to find acceptable and varying forms of PA that works within women’s lifestyle, time, space, and access available, once pelvic floor health is restored.

#### Healthcare, charities, NDPP need to…

8) Support pelvic floor health, which could evolve into further PA longevity as a transition. This could involve linking with exercise specialists or exercise referral programs, who could work directly with women to provide support.
9) Connect and link women after GDM to local and community-based resources and PA opportunities.

### Community

#### Charities, NDPP, family hubs and local PA opportunities need to…

10) Provide free (or cheap)- at-point-of-access PA opportunities within communities and/or locally based (by neighbourhood or locality, through children centres/family hubs, religious settings, sports teams etc.).
11) Co-locate PA opportunities for women in child-focused spaces e.g. children’s sports clubs, in family hub settings etc.
12) Form support groups and connect women after GDM for peer support to enable exercise through:
  - exercising together with their babies/children and.
  - through keeping each other accountable for exercise and PA.

### Policy

Policies to enable recommendations at the social, organisational and community level are needed. For example, policies that allow for subsidising costs and enabling free (or cheap)- at-point-of-access locally based PA opportunities. Policies that can enhance existing pathways and allow for changes to improve and optimise support for women after GDM are needed.

#### Enhancing pathways that exist by…

13) Improving continuity of care and transition back into primary care after birth.
14) Linking to charities, social prescribers and other professionals to link with community and existing resources.
15) Allowing the antenatal team to make referrals related to postnatal support, including to the NDPP and/or exercise referral schemes.
16) Adapting the NDPP so it is accessible and appropriate for women who have had GDM and have young children.

#### Changes to the system require a dedicated…

17) Follow-up point after GDM after 3-months postpartum when women feel ready, for the transition away from GDM into discussions and introduction to long term diabetes risk management.
18) Role within healthcare contexts to PA and supporting lifestyle behaviours, e.g. exercise physiologists, social prescribers. Women after GDM should be linked to these professionals.

*Numbers related to how listed in ‘Final recommendations’ section. Adapted McLeroy et al.*,^*20*^.

## Discussion

This study aimed to generate theory-based pragmatic recommendations to optimise engagement with PA for women with previous GDM. Overall, the results suggest that multiple levels of recommendations must be implemented, as on their own, each recommendation is not the sole solution for many women. Additionally, these recommendations align well with Sport England’s implementation plan for 2022–2025, ‘Meeting people where they’re at’, the ‘better birth report’ by the NHS, and the national response for reducing T2DM risk [34, 35]. For example, the need to work with organisations and communities to transform places to enable PA, the need for community hubs and improvements in postnatal care services and working with various charities and organisations respectively. This further highlights the intersectional needs for women who have had GDM. Below, some of the key points relating to the recommendations are discussed in more detail.

*Behaviour change techniques and educating around T2DM risk are essential but must be addressed alongside multi-level approaches*.

While incorporating BCTs in interventions has been proposed as helpful way to address capability and motivation, a review of BCTs for postpartum women showed no PA changes despite being effective for reducing energy intake [9, 36]. Specifically, the BCT’s included and discussed in this work were related to educating about diabetes risk, goal setting, motivational interviewing, self-monitoring, using reminders and providing feedback [29]. Additionally, the need to educate women about T2DM risk to enhance their motivation for behaviour change has previously been recognised [37]. However, this study and other recent studies suggest that future risk of T2DM is not always a deciding factor for women engaging with PA^38,39^. Chater and Loewenstein argue that focusing solely on individual behaviour change, while cheaper and easier, is insufficient and results in modest changes, and while systemic interventions may be more complex, they could also be more valuable [15]. Therefore, while incorporating BCTs and educating on future T2DM risk is important, this must be done in conjunction with system-level targets of barriers to PA, discussed further below.

### The need for co-located childcare

A lack of childcare is a widely recognised barrier to PA for postpartum women, including those with previous GDM [40, 41]. The lack of available flexible and affordable childcare to enable PA was further discussed in this study. While the need for partner support, and reliance on wider family was an important consideration, this was not suitable for many women who did not have this as an option. Therefore, to enable women to embrace PA, other system-level actors need to step in and create such opportunities, which could benefit all women with young children, and not exclusively those with a history of GDM. Specifically, a key finding of this study was the need for co-locating childcare opportunities within PA spaces. On-site crèche facilities are available across the UK in a limited number of gyms, to which memberships may be high in cost (e.g. a gym in the north of England) thus unattainable for many women [42, 43]. However, while co-located childcare opportunities were a key finding in this work, evidence is still limited, and as highlighted above, practical challenges relating to cost and implementation are still not resolved nor in scope of this paper. Consequently, future research and evaluation of co-located childcare opportunities in PA spaces is necessary, to better understand their potential acceptability and usefulness in enabling PA.

### Capitalising on locally based PA

In addition to a general lack of PA spaces that are accommodating to having young children, this study further highlighted a gap in available PA for women with more than one child, or those with children aged 1–5 years old. Further research is needed to better understand how to address this problem, however, connecting with existing community resources or funding new ones could be one potential solution. For example, results in this study suggest implementing PA opportunities through children’s centres, like hosting accredited exercise professionals to run sessions. In the UK, some local authorities have already received government funding for Family Hubs, a ‘one-stop shop’ to support families through a range of services [44]. An example of a PA based opportunity offered at Family Hubs is a slow ‘Walk and Talk’ session [45]. Evaluations of these opportunities and further research to explore how to integrate PA and co-located childcare within family hubs is warranted, especially as family hubs may operate differently across local authorities [46]. Furthermore, this study discovered that while some community resources may exist, there was a lack of awareness and connection to these. This further calls attention to the need to raise awareness amongst HCPs along the GDM pathways for local or community-based PA opportunities to which women can be directed.

### Changing messages within healthcare

This study found an emphasis on diet and weight-management for reducing risk of T2DM after GDM to have variable outcomes. Wider literature supports a weight focus, given that weight management is highly effective for reducing risk of T2DM after GDM [47–49]. However, for women who find a weight focus demotivating, like when their weight stagnates, this may instead reduce PA engagement. Given that PA may independently reduce risk of T2DM, and that PA focused interventions can effectively increase PA postnatally while weight management interventions do not, a PA focus instead of a weight focus may be more helpful [6, 50]. Additionally, there are likely parallels between the stigma women with and after GDM feel around GDM and lifestyle behaviours, and weight stigma [51, 52]. The impacts of weight stigma within maternal healthcare resulting in poorer health behaviours has also been further emphasised in a recent narrative review [53]. Further research is still needed to explore the effectiveness, acceptability, and implications of PA focused interventions and support for PA uptake and subsequent risk of T2DM after GDM.

### Improving continuity of care

In agreement with this study, previous work has highlighted that a lack of information sharing could contribute to the overlooking of a previous GDM diagnosis in primary care, and that 23.4% of women with previous GDM received no follow-up after delivery, with professionals unsure who is responsible [54, 55]. Therefore, improvement in continuity of care is a fundamental need which, specifically for women after GDM, could allow for a postnatal contact point to support the transition away from a GDM pregnancy and into long-term T2DM prevention. For example, this could be a good opportunity to link and direct women to the NDPP and further PA opportunities and resources. This is especially important, since women after GDM feel unsupported postnatally, and call for increased support [30, 40]. In terms of supporting longer-term T2DM prevention, previous work suggests the need to intervene within the first 6 years, as early as possible due to increased risk of T2DM [56–58]. Maindal et al.,^59^ have more recently suggested initiating 3-months postnatally. In reality, individual preferences and birth experience may influence readiness for this, and it is likely that pragmatic constraints may influence the optimal timing to embed such support within the care pathways [31]. Therefore, while a tailored approach is beneficial, future research should establish whose professional responsibility it would be to oversee this check in, what it should entail, and at what timepoint it could realistically be embedded in the care pathway, given constraints and resources in staff time. Additionally, future work should investigate the cost-effectiveness of such a change in the care pathway, to support applications for funding to improve women’s health.

### Reflections of the use of the SEM

As the work progressed, it became apparent how complex and messy life and interventions to optimise PA are. The SEM is a flexible framework; however, it is still difficult to fully account for the complexities of systems thinking. In this work, the authors did try to account for this in some way, for example by using the SEM to understand interrelationships between factors impacting PA. This pragmatic decision was helpful and aided the dissemination of results. However, even in writing up and as is stated throughout this manuscript, the complexities have had to be simplified, as in reality any one solution at any one level is not the sole solution. Yet, given discussions with the advisory group and other stakeholders when finalising the recommendations, use of the SEM in this way was perceived as helpful for targets going forward, highlighting the complexity and need for a multi-system approach. Nevertheless, a deeper exploration of the complexities captured using the SEM here is still warranted, particularly to validate the effectiveness and sustainability of the system-level recommendations presented.

### Strengths and limitations

The work presented here has several strengths. Firstly, reflexivity was supported in this work through several iterative rounds of refinement, in addition to discussions amongst the authors and advisory group. The combination of reflection, theory generation and use of the SEM as a framework enhances the trustworthiness of the data [60]. Specifically, the use of the SEM to scaffold theories supported the coherence, quality and transparency of the realist-inspired methods [26] and highlighted the complexities of the ‘systems’ surrounding women after GDM. Secondly, theory was generated and tested using different methods, which adds to the robustness of the theory and is productive for realist-inspired theory generation [61]. For example, quantitative data was collected in the first cycle of refinement to understand outcomes, while qualitative data collected in the second cycle of refinement added to the richness and understanding of these outcomes (Fig. 2). Nevertheless, the theory is limited based on the best available evidence to date, so it should be noted that as more evidence become available, these theories may change. Additionally, as the work is limited based on the evidence to date, it does not address the needs of women from diverse ethnic and socioeconomic backgrounds. The overall gaps in equity of evidence to reduce risk of T2DM after GDM is further highlighted in a recent systematic review [62]. Therefore, the recommendations produced do not account for a range of potential inequalities and different needs. Future research should work towards improving representation within this field, purposefully directing resources and time to work with a range of underserved groups. Without this emphasis going forward, the reach and impact of programs aiming to support PA for underserved women will not be equitable, nor effective.

## Conclusion

Pragmatically adapting realist methods to better understand how to optimise physical activity after gestational diabetes was helpful. Policy makers, local authorities and stakeholders involved in intervention design and healthcare provision can use these robust theory-driven recommendations to improve care and support needed for women to engage with activity after gestational diabetes. Specifically, continuity of care and foundational improvements in the pathway women with gestational diabetes enter are needed, in addition to better connecting with organisations and community-based places to do so.

## Data Availability

The datasets used and/or analysed during the current study are available from the corresponding author on reasonable request. However, these cannot be provided if anonymity would be compromised. Published synthesis of data at various stages23-26 that contributed to the work in this manuscript is also available and referenced appropriately in-text.

## Abbreviations

GDM: Gestational Diabetes Mellitus
PA: Physical Activity
T2DM: Type 2 Diabetes Mellitus
HCPs: Health Care Professionals
NDPP: National Diabetes Prevention Program
DUK: Diabetes UK
BCTs: Behaviour Change Techniques including educating about diabetes risk, goal setting, motivational interviewing, self-monitoring, using reminders and providing feedback
WHO: World Health Organization
RAMESES: Realist And Meta-narrative Evidence Syntheses: Evolving Standards
SEM: Socio-Ecological Model
COVID19: Coronavirus Disease 2019
NIHR: National Institute for Health and Care Research
GP: General Practitioner
